# Open versus robotic-assisted techniques for multivisceral pelvic resections of locally advanced or recurrent colorectal and anal cancers: short-term outcomes from a single centre

**DOI:** 10.1007/s10151-024-03044-9

**Published:** 2024-11-19

**Authors:** J. Wyatt, E. O’Connell, M. Choi, S. G. Powell, V. Hanchanale, S. Ahmed, M. A. Javed

**Affiliations:** 1 Department of Colorectal Surgery, Liverpool University Hospitals NHS Foundation Trust, Liverpool, L7 8XP UK; 2 Institute of Life Course and Medical Sciences, University of Liverpool, Liverpool, L1 8JX UK; 3 Institute of Systems, Molecular and Integrative Biology, University of Liverpool, Liverpool, L1 8JX UK

**Keywords:** Pelvic exenteration, Robotic-assisted surgery, Multivisceral pelvic surgery, Minimally invasive surgery, Resection margin

## Abstract

**Background:**

Pelvic exenterations are now established as a standard of care for locally advanced and recurrent rectal cancer. Traditionally, these radical and complex operations have been performed via an open approach, but with the increasing expertise in robotic-assisted surgery (RAS), there is scope to perform such cases robotically. This study compares outcomes from open and RAS pelvic exenterations.

**Methods:**

This retrospective observational study includes all pelvic exenterations for locally advanced or recurrent colorectal cancers performed in a single centre between September 2018 and September 2023. Cases were grouped into open or RAS surgery and classified in terms of operative extent and complexity. The primary outcome was resection margin status. Secondary outcomes were postoperative morbidity, length of stay and blood loss.

**Results:**

Thirty-three patients were included. Nineteen (57.6%) cases utilised an open technique, and 14 (42.4%) used RAS. Patient characteristics and operative complexity were equivalent between groups. R0 rate (63.1% vs 71.4%, *p* = 0.719), median haemoglobin drop (19 (11–30) g/L vs 13 (5–26) g/L, *p* = 0.208) and postoperative morbidity (18/19 (94.7%) vs 9/14 (64.3%), *p* = 0.062) were equivalent. Length of stay (16.0 days (8–25) vs 9.5 days (6–16), *p* = 0.047) was shorter in the RAS group.

**Conclusions:**

Short-term surgical and histopathological outcomes are equivalent in this small cohort of patients. This study suggests that RAS may be a safe and effective method for performing pelvic exenterations for colorectal malignancies. Larger-scale and robustly designed prospective studies are required to confirm these preliminary findings and report on long-term oncological outcomes.

## Introduction

Over the last 10–15 years, pelvic exenterations have been established as a curative-intent standard of care for recurrent and locally advanced colorectal cancers [1]. As such, there has been a global increase in clinical centres providing this service [2]. However, this radical surgical approach is associated with high levels of morbidity and a significant impact on patient quality of life [3, 4]. Therefore, there is a clinical need to explore methods and techniques to limit the adverse consequences of exenterative surgery.

Minimally invasive surgery in the form of both laparoscopic and robotic-assisted surgery (RAS) is well established in colorectal practice and provides a benefit to short-term postoperative morbidity, length of stay, and pain [5, 6]. RAS, in particular, may lend itself to the complex nature of pelvic dissection, and some centres are starting to report case series and small observational studies utilising RAS for multivisceral pelvic resections [7–11]. A 2018 systematic review from the global PelvEx collaborative summarised the available data and demonstrated reduced blood loss, length and stay and overall morbidity in selected patients [12]. However, the novelty of this approach means data remains limited and large-scale studies are non-existent.

Furthermore, pelvic exenterations incorporate a heterogeneous group of procedures with varying complexity and extent. Previous publications demonstrate the early feasibility of RAS but provide little evidence of stratification by surgical extent. The recently published pelvic exenteration lexicon [13] enables this, but there is an ongoing need for evidence, stratified by complexity, regarding the safety and efficacy of RAS in pelvic exenterative surgery.

This study aims to compare short-term outcomes between open and RAS pelvic exenterations within a single, high-volume tertiary centre with experienced RAS surgeons.

## Methods

This retrospective observational study is reported in accordance with the Strengthening the Reporting of Observational Studies in Epidemiology (STROBE) guidelines [14]. Local approvals for data collection were sought and provided.

### Study design and eligibility criteria

All adult patients who underwent elective, curative-intent, resectional surgery extending beyond conventional surgical planes and/or resections of multiple pelvic viscera were included. All operations were conducted at a single tertiary referral centre (Liverpool University Hospitals NHS Foundation Trust, UK) between September 2018 and September 2023. Indications for surgery included both locally advanced or recurrent colorectal adenocarcinoma and recurrent squamous cell cancer of the anal canal.

Patients were divided into two groups. The first was patients whose intra-abdominal component of surgery was completed using a robotic-assisted approach, and the second was those whose surgery was performed using an entirely open approach.

### Preoperative pathway

All patients were discussed in the relevant multidisciplinary team (MDT) meeting and were subject to conventional preoperative computed tomography (CT) and pelvic magnetic resonance imaging (MRI) staging. Positron-emission tomography (PET-CT) was used selectively to assess for additional sites of disease.

### Surgical technique

Robotic-assisted resections were conducted using the DaVinci X or Xi surgical systems (Intuitive Surgical Inc., Sunnyvale, USA), with all intra-abdominal components performed without conversion to an open operation. Gynaecological, urological, spinal orthopaedic and reconstructive plastic surgeons were involved when required, with each case led by a colorectal surgeon. All participating surgeons had a specialist interest in complex pelvic surgical oncology.

Decision-making regarding the operative approach was based on the individual surgeon’s preference and skill set. Over the course of the study period, there was a trend toward a RAS approach as surgeons’ expertise in this modality increased. No traditional laparoscopic procedures were performed during the study period, and no procedures meeting the eligibility criteria were excluded.

### Data collection

Patients were identified through a retrospective search of theatre and MDT meeting logs. All relevant patient records were reviewed, and the following data points were extracted to a purpose-designed spreadsheet: Patient demographics, tumour histology and staging, neoadjuvant therapy, extent and method of surgical intervention, postoperative morbidity and mortality, perioperative haematological and biochemical markers, length and location of inpatient stay, resection margin status, and short-term survival.

The Clavien-Dindo (CD) classification system was utilised to classify postoperative morbidity [15]. Given the heterogeneity in the extent and complexity of multivisceral pelvic resections, all operations were classified according to the ‘pelvic exenteration lexicon’ [13]. The recently published lexicon advances prior attempts to classify exenterations [16] and provides a framework for objective classification and more accurate comparisons across studies. In accordance with this publication, all operations were defined as either conventional or high complexity, and each operation was coded according to the specific organs and tissues resected. Conventional complexity is defined as a “procedure where all or most organs in the pelvic cavity are removed”. High complexity is conventional exenteration plus “surgery to remove bony structures or the structures in the pelvic sidewall” [13].

### Outcome measures

The primary outcome was resection margin status. Resection margins were defined as positive (R1) if tumour cells were found at or within 1 mm of the resection margin at the time of histopathological assessment [17].

Secondary outcomes included length of stay, postoperative morbidity as defined by CD complication grade and drop in haemoglobin at day 1 postoperatively.

### Statistical analysis

Statistical analyses were performed using the STATA software (Ver 18.0, Texas, USA). Continuous variables were reported as medians with the associated interquartile range (IQR), and categorical variables were reported as whole numbers and percentages.

Patient characteristics and outcomes were compared between groups using the non-parametric Mann–Whitney* U* test for continuous variables and the chi-square (χ^2^) test for categorical variables. Where categorical values had less than 10 cases in a group, the Fisher exact test was used instead. *p* values < 0.05 were considered statistically significant.

## Results

A total of 33 patients who met the eligibility criteria and underwent surgery between September 2018 and September 2023 were included in this study. Nineteen (57.6%) patients underwent an open operation, and 14 (42.4%) underwent a robotic-assisted operation.

### Patient and tumour characteristics

Overall and grouped patient and tumour characteristics are summarised in Table 1. The median age of the patients included was 65.5 (57–69), and 17 (51.5%) patients were male. Twenty (60.6%) patients had an American Society of Anaesthesiology (ASA) score of 1–2, and 13 (39.4%) had a score of 3–4. Body mass index (BMI) tended towards normal or underweight patients. Three (9.1%) patients had a BMI greater than 35.

**Table 1:** Patient and tumour characteristics

	All patients	Open	Robotic	*p* value
	(*n* = 33)	(*n* = 19)	(*n* = 14)	
Age, years, median (IQR)	65.5 (57–69)	63 (56–69)	67 (59–70)	0.409
Age categorised, years,* n* (%)				
< 65	16 (48.5)	10 (52.6)	6 (42.9)	0.643
65–75	14 (42.4)	8 (42.1)	6 (42.9)	
> 75	3 (9.1)	1 (5.3)	2 (14.3)	
Gender,* n* (%)				
Male	17 (51.5)	9 (47.4)	8 (57.1)	0.579
Female	16 (48.5)	10 (52.6)	6 (42.9)	
ASA score, *n* (%)				
I–II	20 (60.6)	13 (68.4)	7 (50.0)	0.284
III–IV	13 (39.4)	6 (31.6)	7 (50.0)	
BMI, kg/m^2^, median (IQR)	24.9 (22.8–28.3)	26.5 (23.4–28.5)	23.49 (21.8–26.8)	0.212
BMI categorised, kg/m^2^, *n* (%)				
< 25	17 (51.5)	8 (42.1)	9 (64.3)	0.451
25–35	13 (39.4)	9 (47.4)	4 (28.6)	
> 35	3 (9.1)	2 (10.5)	1 (7.1)	
Presentation, *n* (%)				
Elective	33 (100)	19 (100)	14 (100)	
Emergency	0	0	0	
Previous open surgery, *n* (%)				
Yes	17 (51.5)	8 (42.1)	9 (64.3)	0.208
No	16 (48.5)	11 (57.9)	5 (35.7)	
Adenocarcinoma pT stage				
1–3	10 (38.5)	7 (46.7)	3 (27.3)	0.376
4a	4 (15.4)	1 (6.7)	3 (27.3)	
4b	12 (46.2)	7 (46.7)	5 (45.5)	
Adenocarcinoma pN stage				
0	9 (34.6)	6 (40.0)	3 (27.3)	0.598
1	11 (42.3)	5 (33.3)	6 (54.5)	
2	6 (23.1)	4 (26.7)	2 (18.2)	
Adenocarcinoma M stage				
0	20 (76.9)	11 (73.3)	9 (81.8)	0.491
1	6 (23.1)	4 (26.7)	2 (18.2)	
SCC pT stage				
1–3	4 (57.1)	1 (25.0)	3 (100.0)	0.114
4a	0 (0.0)	0 (0.0)	0 (0.0)	
4b	3 (42.9)	3 (75.0)	0 (0.0)	
SCC pN stage				
0	3 (42.9)	2 (50.0)	1 (33.3)	1.00
1	3 (42.9)	1 (25.0)	2 (66.7)	
2	1 (14.3)	1 (25.0)	0 (0.0)	
SCC M stage				
0	7 (100.0)	4 (100.0)	3 (100.0)	
1	0 (0.0)	0 (0.0)	0 (0.0)	

*SD* standard deviation, *ASA* American Society of Anaesthesiologists, *BMI* body mass index, *kg* kilogram, *IQR* interquartile mean, *SCC* squamous cell carcinoma

All patients were operated on in an elective setting. Seventeen (51.5%) had undergone previous major open surgery, including nine (64.3%) patients in the RAS group. There was no statistical difference between demographics of the two groups. There were seven (21.2%) cases of squamous cell carcinoma, and the remainder (26 (78.8%)) were adenocarcinomas (*p* = 0.401). Postoperative, histological T and N staging was similar between groups and tended towards more advanced tumours, with 19 (57.6%) staged as pT4a/b.

### Patient outcomes

Table 2 summarises the overall and grouped outcomes. Resection margins were negative (R0) in 22 (66.7%) cases and positive (R1) in 11 (33.3%). There was no difference in R0 resection rate between open and RAS groups (12 (63.1%) and 10 (71.4%) cases, respectively (*p* = 0.719)). There were no cases of R2 resection margins.

**Table 2:** Outcomes

	All patients (*n* = 33)	Open (*n* = 19)	Robotic (*n* = 14)	*p* value
CD complication, *n *(%)				
None	7 (21.2)	2 (10.5)	5 (35.7)	0.196
Minor (I–II)	18 (54.5)	11 (55.0)	7 (53.8)	
Major (III–IV)	8 (24.2)	6 (31.6)	2 (14.3)	
Any (I–IV)	26 (78.8)	17 (89.5)	9 (64.3)	0.120
Hb drop day 1, g/L, median (IQR)	17 (9–29)	19 (11–30)	13 (5–26)	0.208
Blood transfusion, *n*(%)	3 (9.1)	3 (15)	0 (0)	0.178
Length of stay, days, median (IQR)	12 (8–22)	16 (8–25)	9.5 (6–16)	0.047
Operative time (mins), median (IQR)	402 (355–469)	355 (278–438)	474 (413–507)	< 0.05
Resection margins, *n *(%)				
R0	22 (66.7)	12 (63.1)	10 (71.4)	0.719
R1	11 (33.3)	7 (36.8)	4 (28.6)	
30-day mortality, *n*(%)	1 (3.0)	1 (5.0)	0 (0.0)	

*CD* Clavien-Dindo, *Hb* haemoglobin, *IQR* interquartile range

The postoperative day 1 haemoglobin level and the requirement for blood transfusion was compared with the preoperative value as surrogate markers of intraoperative blood loss. Open operations were associated with a median drop of 19.0 g/L compared to 13.0 g/L in the RAS group (*p* = 0.208). Operative time was significantly longer in the RAS group with a median of 474 min versus 355 min in the open group (*p* < 0.05). Three patients needed a blood transfusion perioperatively, all within the open group (*p* = 0.178). Median length of stay was 16 days in the open group and 9.5 days in the RAS group (*p* = 0.047). In the open group, 17 (85.0%) patients had a postoperation complication of any severity compared to nine (64.3%) in the robotic group (*p* = 0.120). Eight grade III–IV complications occurred, six of which were in the open group (30.0%) and included four returns to theatre for anastomotic or stump leaks, one interventional radiological (IR) drain insertion for abdominal collection and one nephrostomy for obstructive uropathy. The two grade III–IV complications in the robotic group (15.4%) comprised a return to theatre for small bowel obstruction at a port site and one IR guided drain insertion for an infective collection. There were no deaths within 90 days of surgery.

### Operation extent and complexity

Table 3 presents the extent of tissue and visceral resection according to the classification of surgical complexity defined by the ‘pelvic exenteration lexicon’. Twenty (60.6%) cases were defined as conventional complexity, and 13 (39.4%) were defined as high complexity. High-complexity cases were evenly distributed between groups, with 8 (42.1%) in the open group and 5 (35.6%) in the robotic group (*p* = 0.485).

**Table 3:** Operative extent and complexity

	All patients (*n* = 33)	Open (*n* = 19)	Robotic (*n* = 14)	*p* value
Complexity of pelvic exenteration				
Conventional	20 (60.6)	11 (57.9)	9 (64.3)	0.485
High complexity	13 (39.4)	8 (42.1)	5 (35.7)	
Central compartment				
C1	19 (57.6)	11 (57.9)	8 (57.1)	1.00
C2	13 (39.4)	8 (42.1)	5 (35.7)	
C3	0 (0.0)	0 (0.0)	0 (0.0)	
Anterior compartment				
A1	5 (15.2)	3 (15.8)	2 (14.3)	0.441
A2	4 (12.1)	4 (21.1)	0 (0.0)	
A3	4 (12.1)	2 (10.5)	2 (14.3)	
A4	0 (0.0)	0 (0.0)	0 (0.0)	
A5	0 (0.0)	0 (0.0)	0 (0.0)	
Posterior compartment				
P1	1 (3.0)	1 (5.3)	0 (0.0)	1.00
P2	0 (0.0)	0 (0.0)	0 (0.0)	
P3	6 (18.2)	4 (21.1)	2 (14.3)	
P4	0 (0.0)	0 (0.0)	0 (0.0)	
P5	0 (0.0)	0 (0.0)	0 (0.0)	
Vessels				
SV1	3 (9.1)	3 (15.8)	0 (0.0)	
SV2	0 (0.0)	0 (0.0)	0 (0.0)	
SV3	0 (0.0)	0 (0.0)	0 (0.0)	
SV4	0 (0.0)	0 (0.0)	0 (0.0)	
Nerves				
SN1	0 (0.0)	0 (0.0)	0 (0.0)	
SN2	0 (0.0)	0 (0.0)	0 (0.0)	
SN3	0 (0.0)	0 (0.0)	0 (0.0)	
SN4	0 (0.0)	0 (0.0)	0 (0.0)	
SN5	0 (0.0)	0 (0.0)	0 (0.0)	
Pelvic floor/muscles				
PM1	2 (6.1)	2 (10.5)	0 (0.0)	
PM2	0 (0.0)	0 (0.0)	0 (0.0)	
PM3	0 (0.0)	0 (0.0)	0 (0.0)	
Reconstruction				
F1	10 (30.3)	6 (31.6)	4 (28.6)	
F2	0 (0.0)	0 (0.0)	0 (0.0)	
Additional				
E1	3 (9.1)	1 (5.3)	2 (14.3)	
E2	0 (0.0)	0 (0.0)	0 (0.0)	
E3	0 (0.0)	0 (0.0)	0 (0.0)	
E4	0 (0.0)	0 (0.0)	0 (0.0)	
E5	0 (0.0)	0 (0.0)	0 (0.0)	

A central compartment resection was performed in 32 (97%) cases, with the remaining one case being recurrent rectal cancer where the central compartment had previously been excised. Thirteen (39.3%) cases included an anterior compartment resection, and seven (21.2%) cases included a posterior compartment resection. Pelvic sidewall vessel resection and pelvic floor musculature resection were performed in three (9.1%) and two (6.1%) cases, respectively. Plastic surgical flap reconstruction of the perineum and pelvic floor was performed in 10 (30.3%) cases. No operations included a pelvic side wall nerve resection.

Analyses of CD grade, Hb drop, length of stay and resection margin status were repeated with cases divided into high and conventional complexity groups to further analyse the impact of operation extent on outcomes. No statistically significant difference was demonstrated between these groups (Table 4). However, a possible trend was seen towards a shorter median length of stay in the conventional complexity group (9 days (7–22)) compared to the higher-complexity group (16 days (10–22), *p* = 0.253).

**Table 4:** Outcomes in standard and high-complexity groups

	All patients (*n* = 33)	High complexity (*n* = 13)	Conventional complexity (*n* = 20)	*p* value
CD complication, *n* (%)				
None	6 (18.2)	2 (15.4)	4 (20.0)	1.000
Minor (I–II)	18 (54.5)	7 (53.8)	11 (55.0)	
Major (III–IV)	9 (27.3)	4 (30.8)	5 (25.0)	
Hb drop day 1, g/L, median (IQR)	17 (9–29)	17 (9–32)	16 (9–24.5)	0.726
Length of stay, days, median (IQR)	12 (8–22)	16 (10–22)	9 (7–22)	0.253
Resection margins, *n* (%)				
R0	22 (66.7)	8 (61.5)	14 (70.0)	0.714
R1	11 (33.3)	5 (38.5)	6 (30.0)	
90-day mortality	0 (0.0)	0 (0.0)	0 (0.0)	

*CD* Clavien-Dindo, *Hb* haemoglobin, *IQR* interquartile range

## Discussion

This single-centre, retrospective, case–control study compares the short-term surgical outcomes in a complex cohort of patients undergoing pelvic exenterations using either an open or robotic-assisted approach. In these groups, with similar patient and tumour characteristics, operative extent and complexity, robotic-assisted operations demonstrate similar oncological and postoperative outcomes to an open approach.

The R0 rate in this study was equivalent between operative approaches. As the most important prognostic marker of survival and quality of life in patients undergoing pelvic exenterations [18, 19], resection margin status is critical and should not be compromised in the pursuit of lower morbidity techniques. R0 rates from RAS exenterations in other centres range widely from 66% to 100% [7–9, 20]. The rates reported here are significantly limited by the small patient population where one or two positive margins substantially lower the overall rate. For RAS to be a viable approach it is imperative that R0 rates are non-inferior to open surgery and larger cohorts are required to further delineate the R0 rate.

Adhesions from previous surgeries are often seen as a barrier to minimally invasive approaches, with some surgeons preferring an open approach in this scenario. The high rate of previous surgery we report in the RAS group suggests that previous surgery alone should not deter the use of RAS, on the basis of our experience.

Morbidity and blood loss were statistically equivalent between the open and RAS groups in this study. However, these outcomes trended towards favouring RAS. It is likely that these outcomes would have reached significance with a greater sample size. Indeed, a similar, higher-volume study of 35 RAS pelvic exenterations and 70 open exenterations reported a similar drop in median length of stay (3 days), blood loss (400mls) and postoperative complication rate (26%) but demonstrated statistical significance in each outcome. We confirm a shorter length of stay in RAS, suggesting quicker postoperative recovery and lower healthcare costs.

Operative time was significantly longer in the RAS group, a finding replicated in multiple studies comparing RAS to both open and laparoscopic approaches in various fields [21–23]. A recent meta-analysis which includes 264 patients undergoing minimally invasive pelvic exenteration confirmed a significantly longer operative time with RAS [24]. Therefore, exenterative surgery appears to be no exception and it is even likely that this disadvantage may be compounded as a result of the inherent complexity of this surgery.

RAS pelvic exenteration represents a relatively novel frontier, with a limited number of centres worldwide reporting on cases conducted using this approach [7, 10, 25]. This scarcity underscores the innovative nature of RAS in this complex field, as well as the need for further exploration and dissemination of expertise. However, although this study demonstrates encouraging outcomes in select cases, the relevance of open surgery in pelvic exenteration persists for compelling reasons. The inherent complexity and variability inherent in these cases necessitates that open surgery continues to be an essential tool in the armamentarium. Although RAS presents a significant advancement in surgical technology, there will invariably be scenarios where open surgery is preferred or required, thus highlighting the complementary roles of both approaches in the contemporary surgical landscape [26].

This study is inherently limited by the retrospective design and small patient population, and it is impossible to state firm conclusions regarding the efficacy of RAS in this setting. Specifically, possible trends in blood loss, complication rate and length of stay did not reach significance and it is possible this is attributable to the population size. All available evidence in this field to date suffers from similar limitations. Therefore, prospective and multicentre studies are required to inform the safety and efficacy of RAS pelvic exenterations, and it is with great interest that we await the outcomes of the ROPES collaborative, which seeks to provide this [27].

A further limitation of this study is the absence of long-term outcomes due to the evolving nature of the RAS programme. A broader, global lack of data regarding long-term oncological outcomes also needs to be addressed. Furthermore, patient-reported outcome measures and quality of life indicators would further guide the relevance of RAS in this context but were not available because of the retrospective nature of this study [28]. Finally, this study is limited by the inclusion of different tumour types and recurrent and primary cancers with varied outcomes and biological behaviour. Specifically, anal squamous cell cancers are included within the study population, but tumour biology should not significantly affect the short-term clinical outcomes reported here. However, this study adds to the weight of evidence suggesting the feasibility of an RAS approach to pelvic exenterations and provides further data for meta-analytical synthesis.

## Conclusions

This study demonstrates that RAS pelvic exenterations have similar histopathological and short-term outcomes compared to open procedures and therefore suggests this approach is safe and feasible. Given the inherent biases of the study design and small patient population, results must be viewed with caution. Large-scale, prospective, multicentre studies are required to demonstrate the efficacy of RAS exenterations. Still, there is a possible benefit for morbidity and length of stay for appropriately selected patients, even if they have had previous abdominal surgery.

## Data Availability

Research data will be made available on reasonable request.
